# Phytochemicals against Osteoarthritis by Inhibiting Apoptosis

**DOI:** 10.3390/molecules29071487

**Published:** 2024-03-27

**Authors:** Hui Kong, Juan-Juan Han, Gorbachev Dmitrii, Xin-an Zhang

**Affiliations:** 1 College of Exercise and Health, Shenyang Sport University, Shenyang 110102, China; 17852060669@163.com (H.K.); hanhan9210@163.com (J.-J.H.); 2 General Hygiene Department, Samara State Medical University, Samara 443000, Russia; d.o.gorbachev@samsmu.ru

**Keywords:** osteoarthritis, phytochemicals, natural products, apoptosis, mechanism

## Abstract

Osteoarthritis (OA) is a chronic joint disease that causes pathological changes in articular cartilage, synovial membrane, or subchondral bone. Conventional treatments for OA include surgical and non-surgical methods. Surgical treatment is suitable for patients in the terminal stage of OA. It is often the last choice because of the associated risks and high cost. Medication of OA mainly includes non-steroidal anti-inflammatory drugs, analgesics, hyaluronic acid, and cortico-steroid anti-inflammatory drugs. However, these drugs often have severe side effects and cannot meet the needs of patients. Therefore, safe and clinically appropriate long-term treatments for OA are urgently needed. Apoptosis is programmed cell death, which is a kind of physiologic cell suicide determined by heredity and conserved by evolution. Inhibition of apoptosis-related pathways has been found to prevent and treat a variety of diseases. Excessive apoptosis can destroy cartilage homeostasis and aggravate the pathological process of OA. Therefore, inhibition of apoptosis-related factors or signaling pathways has become an effective means to treat OA. Phytochemicals are active ingredients from plants, and it has been found that phytochemicals can play an important role in the prevention and treatment of OA by inhibiting apoptosis. We summarize preclinical and clinical studies of phytochemicals for the treatment of OA by inhibiting apoptosis. The results show that phytochemicals can treat OA by targeting apoptosis-related pathways. On the basis of improving some phytochemicals with low bioavailability, poor water solubility, and high toxicity by nanotechnology-based drug delivery systems, and at the same time undergoing strict clinical and pharmacological tests, phytochemicals can be used as a potential therapeutic drug for OA and may be applied in clinical settings.

## 1. Introduction

Osteoarthritis (OA) is a whole joint disease involving all joint tissues: cartilage and meniscal degeneration, inflammation and fibrosis of the infrapatellar fat pad and synovial membrane, and subchondral bone remodeling [1]. It is reported that, in 2019, China had the largest number of OA cases, followed by India and the United States. The prevalence of OA is related to gender and age. The prevalence of OA among women in the world is significantly higher than that of men. In 2021, >22% of adults older than 40 had knee OA. With an increase in age, the prevalence of OA gradually increases, reaching a peak at the age of 60–64 [2]. It is estimated that over 500 million individuals are currently affected by OA worldwide [3]. It is estimated that, by 2032, the global OA prevalence rate will rise from 26.6% to 29.5% [4]. The progress of OA is also related to some bad habits, such as a high-sugar diet and smoking [5]. Different clinical symptoms will appear in different stages of OA. The main symptoms of acute stage OA are pain and swelling. If it is not treated in time, the condition may progress to muscle atrophy, joint stiffness, and other symptoms in the recovery stage [6]. These symptoms will not only seriously affect the daily life and work of patients with OA but also cause negative emotions, such as depression and anxiety [7]. The pathological mechanism of OA mainly includes extracellular matrix degradation, apoptosis, autophagy, and inflammation [8].

Apoptosis is programmed cell death, which refers to physiological cell suicide determined by heredity and conserved in evolution [9]. Apoptosis starts by activating intracellular caspase. The contents of dead cells are wrapped by functional intact cell membranes to form apoptotic bodies, which are then swallowed by other cells. It usually involves no omission or inflammation of the contents of dead cells [10]. In eukaryotes, apoptosis plays a vital role in the development and stability of the organism itself. When the apoptosis regulation mechanism is imbalanced, it can cause insufficient or excessive apoptosis, leading to related diseases, such as tumors and autoimmune diseases [11]. Excessive apoptosis can cause cardiovascular diseases, viral infections, neurodegenerative diseases, and articular cartilage degeneration [12]. In the physiological state, chondrocyte proliferation and apoptosis are in a dynamic balance to maintain the stability of cell number and function. However, this dynamic balance is broken if chondrocytes undergo excessive apoptosis. Studies have shown that chondrocyte apoptosis plays an essential role in the pathological process of OA and is expected to become an important target for OA treatment [13].

Recent studies found that phytochemicals are essential in preventing and treating diseases by inhibiting apoptosis [14]. Phytochemicals are formed by directionally collecting and concentrating one or more active ingredients in plants without changing the structure of active ingredients. They are natural and safe products used for health care, and they have the ability to relieve some diseases [15]. In recent years, phytochemicals have been widely used to prevent and treat many diseases and are also widely used in OA. Many studies have found that phytochemicals can inhibit apoptosis through different mechanisms and play an essential role in preventing or treating OA [16]. However, no comprehensive review article has summarized how phytochemicals can improve OA by mediating apoptosis-related pathways. This paper summarizes phytochemicals from different plants, which may enhance articular cartilage by regulating apoptosis-related signaling pathways and finally treat OA. We use the authoritative internet databases of PubMed, Web of Science, and Embase to systematically search the scientific literature for the last decade, combining the keywords “apoptosis”, “osteoarthritis”, and “plants”. The main search criterion is to use phytochemicals from different plants to treat OA by inhibiting apoptosis. These phytochemicals can be divided into polyphenols, flavonoids, terpenoids, and small molecular compounds. The species and family names of all phytochemicals mentioned in this review are based on http://mpns.kew.org/mpns-portal/, http://www.plantsoftheworldonline.org, accessed on 12 December 2023.

## 2. Apoptosis and OA

Under physiological conditions, apoptosis is a normal process. However, if it is excessive, it becomes a pathological process. Excessive apoptosis exists in many diseases, such as Alzheimer’s [17], cardiovascular disease [18], and cancer [19]. In recent years, many studies have proven that chondrocyte apoptosis may significantly influence the pathogenesis of OA.

Members of the caspase family, Bax, and Bcl2, are critical apoptosis factors. During an acute attack of OA, pro-inflammatory cytokines interleukin-1beta (IL-1β) and tumor necrosis factor alpha (TNF-α) are highly expressed, which upregulate the expression of acid-sensitive ion channel 1a (ASIC1a) in articular chondrocytes by activating the nuclear factor-kappa B (NF-κB) signaling pathway, reduce the survival rate of chondrocytes stimulated by acid, promote the expression of caspase 3 and caspase 9, and, finally, aggravate OA [20]. IL-1β and TNF-α stimulation can also increase the expression of dynamin-related protein 1 (DRP1), which can induce oligomerization of Bax and promote the mitochondrial translocation of Bax. In addition, extracellular signal-related kinases 1 and 2 (ERK1/2) are activated in OA, and ERK1/2 is the activator of DRP1. ERK1/2 can promote DRP1 activation and mitochondrial translocation by activating DRP1 and inducing apoptosis [21].

Many cytokines, non-coding RNA, and compounds can regulate apoptosis in various ways, thereby alleviating the pathological process of OA. Methyltransferase-like 3 (Mettl3) is anm6A methylase, which may regulate the stability of Bcl2 by binding to Bcl2 mRNA. Overexpression of Mettl3 can significantly increase the methylation level of m6A, increase the expression of Bcl2, and inhibit the apoptosis of chondrocytes induced by TNF-α [22]. CircFOXO3 can promote the expression of Bcl2, inhibit the expression of caspase 3 and Bax, and slow down the apoptosis of chondrocytes caused by OA inflammation by regulating FOXO3 [23]. In OA, the small molecular compound XMU-MP-1 can increase mammalian sterile 20-like kinase 1 (MST1) and yes-associated protein (YAP). When YAP is knocked down, apoptosis increases. XMU-MP-1 regulates chondrocyte apoptosis in a YAP-dependent way to slow down cartilage degeneration and alleviate OA.

**Figure 1:**
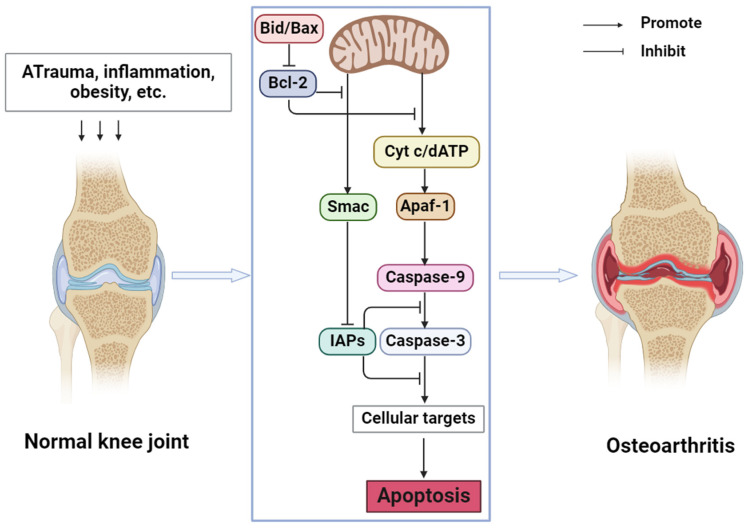
Mechanism of apoptosis in OA (Abbreviations: Bid, BH3-interacting domain death agonist; Bax, Bcl-2-associated X protein; Bcl-2, B cell lymphoma-2; cyt c, cytochrome c; dATP, deoxyadenosine triphosphate; Smac, second mitochondrial-derived activators of caspases; IAPs, Inhibitors of Apoptosis Proteins; Apaf-1, Apoptotic Protease Activating Factor-1).

In OA, the NF-κB signaling pathway, Wnt signaling pathway, and mitogen-activated protein kinases (MAPK) signaling pathway are closely related to apoptosis. Guo et al. [24] found that STING overexpression promotes the expression of Bax, Cytochrome c (cyt c), and caspase 3 and decreases the expression of Bcl2. At the same time, STING knockdown may alleviate the apoptosis induced by IL-1β. Inflammatory cytokines in OA may cause DNA damage in cells. Cytoplasmic DNA may activate STING and further activate the NF-κB signaling pathway so that STING may regulate chondrocyte aging and apoptosis through the NF-κB signaling pathway. MiR-214-3p can not only directly target the Wnt signaling pathway [25] but also inhibit the NF-κB signaling pathway, inhibit apoptosis, and improve OA by targeting IKKβ [26]. In summary, chondrocyte apoptosis is involved in the occurrence and development of OA and plays an indispensable role in it. By regulating chondrocyte apoptosis, joint degeneration can be alleviated and OA can be prevented. The mechanism of apoptosis in OA is shown in Figure 1.

## 3. Phytochemicals for the Treatment of OA by Inhibiting Apoptosis

**Figure 2:**
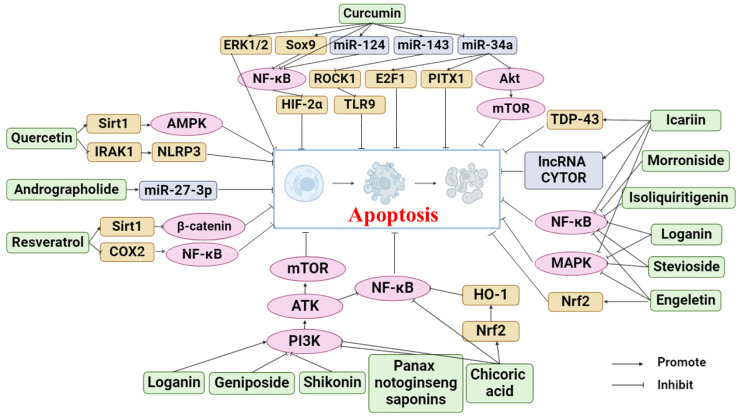
Apoptosis pathways of phytochemicals in anti-OA (Abbreviations: ERK1/2, extracellular regulated kinase1/2; Sox9, recombinant sex-determining region Y box protein 9; ROCK1, rho-associated coiled-coil-containing protein kinase 1; E2F1, E2F transcription factor 1; PITX1, pituitary homeobox 1; HIF-2α, hypoxia-inducible factors-2alpha; TLR9, Toll-Like Receptor 9; PI3K, phosphatidylinositol-3 kinase; Akt, protein kinase B; NF-κB, nuclear factor κB; Sirt1, Sirtuin 1; AMPK, AMP-activated protein kinase; IRAK1, interleukin-1 receptor-associated kinase; NLRP3, NLR family pyrin domain containing 3; COX2, cyclooxygenase-2; TDP-43, TAR DNA binding protein 43; MAPK, mitogen-activated protein kinases; Nrf2, NF-E2-related factor 2; mTOR, mechanistic target of rapamycin; HO-1, heme oxygenase-1).

Phytochemicals are active and healthy substances in plants. Many phytochemicals come from botanical drugs, which are cheap and widely used, and have good pharmacological activities [27]. In recent years, phytochemicals have been found to prevent and treat various diseases, such as cancer [28], cardiovascular diseases [29], and osteoporosis [30], by regulating apoptosis. Many studies have also proven that phytochemicals can target the apoptosis pathway as a supplement and alternative medicine to treat OA. Research on the mechanism by which phytochemicals treat OA through the apoptosis pathway is becoming increasingly extensive. This review summarized the phytochemicals that can treat OA by inhibiting apoptosis, as shown in Table 1. The following sections will describe the anti-OA activities of these phytochemicals and their mechanism. Figure 2 illustrates the specific mechanism of phytochemicals to prevent and alleviate OA by inhibiting apoptosis.

### 3.1. Curcumin

Curcumin [*Zingiberaceae*; *Curcuma longa* L.] (CUR) is a polyphenol compound with anti-inflammatory and antioxidant functions, and it can improve immunity [69]. It can also prevent and treat age-related and aging-related diseases, such as hypertension, chronic respiratory diseases, and OA [70]. CUR may have a good prospect in treating OA by inhibiting apoptosis.

In vivo, CUR can improve cartilage damage in the OA model by inhibiting apoptosis. Yao et al. [37] injected CUR into the articular cavity of rats induced with OA via a high-fat diet. Through WB, RT-PCR, histological analysis, and apoptosis determination, they found that CUR treatment reduces the level of miR-34a and upregulates E2F transcription factor 1 (E2F1) and pituitary homeobox 1 (PITX1). At the same time, the apoptosis of cartilage in OA rats was reduced, thereby alleviating OA-like lesions in rats. It is commendable that the oral bioavailability of curcumin is only 1%, so this study adopts intra-articular injection to improve the bioavailability of curcumin.

In addition, in vitro, some studies have found that CUR can treat OA by mediating the apoptosis-related pathways, thereby improving the status of chondrocytes in the OA model. Qiu et al. [33] treated primary chondrocytes of mice with CUR. Through WB, flow cytometry, immunohistochemistry, luciferase assay, and TUNEL staining, it was found that exosomes of mesenchymal stem cells treated with CUR maintain the vitality of primary chondrocytes, reduce the DNA methylation of miR-143 and miR-124 promoters, further inhibit the expression of their target genes rho-associated coiled-coil-containing protein kinase 1 (ROCK1) and NF-kB, inhibit cell apoptosis, and alleviate the pathological process of OA. Buhrmann et al. [31] used human primary chondrocytes as the research object and treated them with CUR. Through CCK8, WB, immunofluorescence, and immunoprecipitation, they found that CUR positively regulates the expression of sex-determining region Y-box 9 (Sox9), directly inhibits the expression of MMP 9 and caspase 3 in chondrocytes by inhibiting the DNA binding of p65-NF-kB, regulates apoptosis, and, finally, relieves OA. Li et al. [71] applied CUR to the primary chondrocytes of rats to explore its effect on OA chondrocytes and determine its mechanism. Through cell activity assay, flow cytometry, TUNEL staining, WB, and Monodansylcadaverine (MDC) Assay, they found that CUR stimulates the phosphorylation of ERK1/2 in chondrocytes in a time-dependent manner and induces autophagy. Moreover, they observed that CUR decreases the expression of caspase 3, increases the expression of Bcl2, inhibits the apoptosis of chondrocytes caused by IL-1β, and improves OA. Although many preclinical studies have proved the biochemical action of CUR, we need randomized double-blinded and placebo-controlled clinical trials to confirm our in vitro findings. At present, clinical-related research is still relatively lacking.

Various preclinical experiments show that curcumin is safe and can be tolerated even at very high doses. Although curcumin has good pharmacological effects and safety, curcumin has not been approved as a drug, and its bioavailability is considered as a potential reason to limit the complete transformation of in vitro benefits into clinical application [72,73]. Curcumin has low oral absorption, biological distribution, and systemic bioavailability [74,75]. Therefore, some research focuses on formulating methods to overcome these problems. These methods include the use of nanoparticles, liposomes, micelles, and phospholipid complexes to improve the bioavailability, absorption, and biological distribution of curcumin [76]. Therefore, there are still some problems to be solved before CUR is applied to clinical settings.

### 3.2. Resveratrol

Resveratrol [*Vitaceae*; *Vitis vinifera* L.] (RES) is a polyphenol compound that exists in various plants. RES has been proven to have a wide range of biological activities, including anti-inflammatory, antibacterial, antioxidant, and anti-aging effects. It is widely used in the prevention and treatment of cardiovascular diseases [77], liver diseases [78], and OA. Many studies on the treatment of OA with RES involve different mechanisms.

Sirtuin 1 (SIRT1) is a longevity gene related to many aging-related diseases, which plays an essential role in regulating cell apoptosis. RES can activate SIRT1 and inhibit the progress of OA [79]. Using MTT, RT-qPCR, WB, and an apoptosis kit, Liu et al. [41] found that adding RES to human chondrocytes can promote the expression of SIRT1 and inhibit the expression of Wnt3a, Wnt5a, Wnt7a, and β-catenin. The expression of some apoptosis factors such as Bax, caspase 3, and caspase 9 will also be inhibited. Therefore, SIRT1 may regulate apoptosis through the Wnt/β-catenin signaling pathway and treat OA. In addition, Lei et al. [80] found, through WB and immunoprecipitation, that RES inhibits NF-kB activity and nitric oxide (NO) production in chondrocytes in a dose-dependent manner by activating SIRT1 and protecting chondrocytes. In addition to regulating the signaling pathway mediated by SIRT1, RES can restrict other signaling pathways that mediate apoptosis. Li et al. [40] treated chondrocytes with different concentrations of RES. They found that RES can increase the expression of chondrogenic markers and prevent chondrocyte apoptosis through the cyclooxygenase-2 (COX-2)/NF-κB pathway in OA, thereby alleviating the progress of OA.

Different doses of RES have various biological activities. Yi et al. [81] exposed chondrocytes to varying concentrations of RES to determine the biosafety concentration of RES. Their results showed that low to moderate concentrations of RES are not toxic to chondrocytes, whereas high doses of RES have significant harmful effects on chondrocytes. In addition, Wang et al. [38] injected high-dose, medium-dose, and low-dose RES into the articular cavity of rabbits induced with OA via surgery. They evaluated cartilage destruction, NO content, and apoptosis using TUNEL staining, histological staining, and a NO detection kit, respectively. Their results showed that RES protects articular cartilage by inhibiting apoptosis and reducing NO production in cells. This protection was enhanced by increasing the RES dose in the range of 10–50 mol/kg. When determining the dose of RES, ensure that the dose is non-toxic and select the quantity with the best protective effect on OA. Because of the low bioavailability of RES, the molecular structure of RES can be changed or combined with other compounds to obtain multi-target RES derivatives. Therefore, future research needs to focus on the synthesis of RES derivatives in order to improve their bioavailability and enhance their efficacy in relation to OA. The pharmacokinetics and toxicity of RES in OA still need to be further explored through extensive clinical trials.

### 3.3. Chicoric Acid

Chicoric acid [*Asteraceae*; *Echinacea purpurea* (L.) Moench] (CA) is a polyphenolic compound extracted from *chicory* and *echinacea*. It is a rare and valuable functional food ingredient without excessive side effects, contraindications, and drug interactions. As a result of its pharmacological effects in regulating glucose and lipid metabolism, CA has been widely used in drugs, nutritional supplements, and healthy foods [82]. It has anti-apoptotic, antioxidant, and anti-inflammatory activities [83]. CA can also prevent and treat OA through anti-apoptotic effects. Qu et al. [42] found that the CA dose-dependent inhibition of the PI3K/AKT and NF-κB signaling pathways enhances the Nrf2/heme oxygenase-1 (HO-1) signaling pathway and reduces TNF-α-induced ROS production and chondrocyte apoptosis. In vivo experiments have shown that CA treatment significantly reduces the expression of Bax and increases the expression of Bcl2 in the MIA-induced articular cartilage of rats, alleviates the degree of apoptosis, and improves OA. Therefore, CA may be a therapeutic agent for the treatment of OA and can be applied to the prevention and treatment of OA through the anti-apoptotic mechanism. There are still some limitations in the study, including the way CA exerts its biological activity and the effect of CA on osteophytes and synovium. These aspects should be stressed and studied in the future. At present, there are few studies on the treatment of OA using CA, and the evidence is insufficient. We must further study CA to explore its pharmacokinetics, bioavailability, and the ability of these compounds to reach a clinical therapeutic concentration, so as to assess its viability as a drug candidate.

### 3.4. Icariin

Icariin [*Berberidaceae*; *Epimedium brevicornu* Maxim] (ICA) is the main active ingredient of *Epimedium*, which is widely known for its anti-inflammatory and antioxidant properties. In the early days, research on ICA mainly focused on enhancing reproductive and anti-aging functions [84]. In recent years, with the progress of modern pharmacological research, we found that ICA has beneficial effects on various diseases, including nervous system diseases, cardiovascular diseases, and musculoskeletal system diseases [85]. Studies have found that ICA can regulate cell apoptosis through multiple mechanisms and play a preventive and therapeutic role in OA. Wang et al. [48] applied different doses of ICA to chondrocytes. They found that ICA promotes the activity of chondrocytes induced via IL-1β, specifically increases the expression of CYTOR in chondrocytes, and inhibits the apoptosis of chondrocytes. CYTOR knockdown reverses the protective effect of ICA on chondrocytes. Therefore, ICA therapy can protect against chondrocyte apoptosis by regulating CYTOR. However, there are some limitations in this study. Only six lncRNAs were selected in this study, and ICAR may play a protective role in OA by regulating other lncRNAs. In addition, this study was only carried out in vitro experiments, and an in vivo model should be constructed to further verify the conclusion.

In addition, the NF-κB signaling pathway and TAR DNA binding protein 43 (TDP-43) signaling pathway are common signaling pathways through which ICA can protect articular cartilage by inhibiting apoptosis in OA. Liu et al. [86] analyzed the target genes and pathways involved in the mechanism of ICA via bioinformatics analysis, and verified them in animal experiments using WB, QPCR, and flow cytometry. KEGG analysis and molecular biological detection showed that ICA could inhibit apoptosis by regulating the NF-κB pathway, thus protecting articular cartilage. Mi et al. [45] determined the best dose of 10 uM for experimental intervention, and the results were detected via WB, ELISA, and flow cytometry. They found that ICA can downregulate the expression of caspase 3 and caspase 9 induced via TNF-α in chondrocytes, reverse the upregulation of p-p65 and the degradation of IκBα, inhibit the expression of NO and reactive oxygen species (ROS), and prevent cell apoptosis and catabolism. Therefore, ICA can inhibit the apoptosis of chondrocytes by inhibiting the NF-κB signaling pathway and alleviating the pathological process of OA. In addition, Huang et al. [46] found that the expression of TDP-43 is upregulated in rat OA chondrocytes induced via collagenase. Through ELISA and the detection of apoptotic cells, it was found that the cell proliferation rate increased after adding 10, 20, and 40 ng/mL ICA, and the expressions of VEGF, HIF-1α, and TDP-43 in synovial tissue decreased. Therefore, ICA can inhibit apoptosis by regulating various cytokines and the TDP-43 signaling pathway, playing a key role in cartilage protection. However, the above research has some limitations. Specific in silico or in vitro models cannot fully prove the anti-apoptosis characteristics of ICA, and more a rigorous experimental design and sufficient experimental evidence are needed to explore the main mechanism of ICA in cell protection. In addition, the pharmacokinetics of ICA show that ICA will be transformed into metabolites such as icaritin, icariside I, icariside II, and desmethylicaritin, which exist in different cells or tissues, thus exerting a series of chemical effects [87]. However, the target protein and mechanism of ICA in anti-apoptosis in OA are still unclear, which is not enough for clinical application.

### 3.5. Quercetin

Quercetin (QCT) is widely distributed in fruits and vegetables, and it is a kind of flavone famous for its antioxidant, antiviral, antibacterial, and anti-inflammatory properties [88]. QCT has attracted wide attention because of its few side effects and high effectiveness, and it plays a vital role in treating aging-related diseases. Pharmacokinetic studies of QCT in the human body show that the oral bioavailability of QCT is very poor. The absorption rate of QCT glucoside is about 3% to 17% [89]. The relatively low bioavailability of QCT can be attributed to its low absorption rate, extensive metabolism, and rapid elimination. Some studies have addressed the low bioavailability of QCT by discussing various drug delivery systems [90,91]. When its bioavailability and solubility are improved, its therapeutic effect will also increase.

QCT has beneficial effects in anti-OA, such as anti-inflammation, anti-apoptosis, and other biological activities. However, the anti-apoptosis mechanism of QCT is complex and intertwined with various signaling pathways. Wang et al. [51] predicted the signaling pathway related to QCT through network pharmacology and found that the MAPK signaling pathway is highly activated in OA chondrocytes. The optimal QCT concentration of 100 µmol/L was used to intervene in vitro, and the expressions of related factors and signal pathways were detected via QPR and WB. The results show that QCT inhibits the apoptosis of chondrocytes induced via IL-1β by downregulating the p38 MAPK signaling pathway. However, further experiments are still needed to verify this conclusion. Li et al. [52] took the chondrocytes of OA rats intervened via QCT as the research object. They evaluated apoptosis using flow cytometry and Hoechst staining and evaluated protein expression via WB, immunohistochemical staining, and immunofluorescence. The results show QCT reduces the degeneration and erosion of articular cartilage, inhibits inflammation and apoptosis, and downregulates the expression levels of interleukin-1 receptor-associated kinase (IRAK1), NOD-like receptor family pyrin domain containing 3 (NLRP3), and caspase 3. When IRAK1 is overexpressed, cell viability is damaged, inflammation is increased, and apoptosis is enhanced. These effects are weakened in injured rat chondrocytes with NLRP3 knockdown. Therefore, QCT can inhibit inflammation and apoptosis by inhibiting IRAK1/NLRP3 signal transduction and alleviating the progress of OA. However, the experimental design of this study is not rigorous enough, and the possible side effects of QCT in rats and the mechanism of the IRAK1/NLRP3 inflammatory pathway affecting the pathogenesis of OA in vivo are not considered.

There are studies exploring the mechanism of QCT in OA via double verification in vivo and in vitro. Feng et al. [49] verified the role and mechanism of QCT in OA through in vivo and in vitro experiments. Through flow cytometry, TUNEL staining, WB, RT-PCR, and histological staining, they found that QCT can alleviate the apoptosis of tert-butyl hydroperoxide (TBHP)-stimulated rat chondrocytes and inhibit the endoplasmic reticulum stress and apoptosis of related chondrocytes in OA rats and OA articular chondrocytes by activating the IRT1/AMPK signaling pathway, thereby alleviating the pathological process of OA. Hu et al. [50] studied the protective effect of QCT on OA through in vivo and in vitro experiments. In vivo, tissue staining and WB showed that the expression levels of transforming growth factor-beta1 (TGF-β1) and transforming growth factor-beta2 (TGF-β2) in synovial fluid and the proportion of M2 macrophages in synovium increase. In vitro, flow cytometry, qRT-PCR, and WB showed that QCT exerts an anti-apoptosis effect by reducing intracellular ROS, restoring mitochondrial membrane potential (MMP), and inhibiting the caspase 3 pathway in apoptotic rat chondrocytes. Intra-articular injection of QCT can alleviate cartilage degradation and chondrocyte apoptosis in rat OA models. Therefore, both the above two studies show that QCT has anti-apoptosis effect on OA chondrocytes through in vivo and in vitro experiments, which is worthy of our reference in future studies and research. In a word, QCT is a safe dietary supplement, which has a wide range of biological effects on animals, depending on the types of subjects and their health level. It is necessary to further study the administration scheme and adjuvant of QCT for clinical application.

### 3.6. Isoliquiritigenin

Isoliquiritigenin [*Fabaceae*; *Glycyrrhiza glabra* L.] (ISL), one of the active components in *Glycyrrhiza*, is a flavonoid with a simple chalcone structure [92]. It has pharmacological effects such as antioxidant, anti-inflammatory, and anti-apoptosis functions. Many studies have been conducted on the use of ISL in cancer treatment, such as pancreatic [93], lung cancers [94], and gallbladder cancers [95]. ISL can also be used in the prevention and treatment of OA through its anti-apoptotic effect. Ji et al. [53] pretreated ATDC5 cells with low, medium, and high concentrations of LSL in vivo and in vitro. Apoptosis factors were detected using WB and flow cytometry, and the results show that the expression level of anti-apoptotic protein Bcl2 increases dose-dependently. In contrast, the expression level of pro-apoptotic protein Bax and the phosphorylation level of NF-κB stimulated by IL-1β decrease dose-dependently. ISL can reduce the apoptosis rate of ATDC5 cells. ISL also plays a protective cartilage role in the knee tissues of mice. Therefore, ISL can prevent IL-1β-induced, chondrocyte-like ATDC5 apoptosis and alleviate OA by inhibiting NF-κB activation. ISL may work at the early pathological change of OA, so it can be used as a potential new preventive therapy for OA. However, the mechanism by which LSL affects the NF-κB pathway in chondrocytes remains unclear and requires further study.

Many studies show that ISL has a strong absorption capacity and strong elimination capacity through different routes of administration, including intravenous injection, subcutaneous injection, and oral administration. The oral absorption rate is more than 90%, so oral administration has become the most advanced application method [96,97]. ISL is rapidly and widely distributed throughout the body after absorption. Generally speaking, poor bioavailability, rapid degradation, rapid metabolism, and systemic elimination are the necessary factors leading to insufficient bioavailability [98]. In order to improve the solubility and enhance its bioavailability and distribution, encapsulated ISL nanoparticles or nano-ISL have been developed. Studies have confirmed various biological activities of ISL in animals and cells, but the molecular mechanism of its action is still unclear, and the exact target protein that binds to ISL has not been determined, which will hinder the further clinical application of ISL. When a drug is used in clinical settings, its safety is particularly important. Unfortunately, there are few toxicological evaluation reports about ISL. Therefore, a lot of research on ISL is still needed before it is applied to clinical settings.

### 3.7. Loganin

Loganin [*Cornaceae*; *Cornus officinalis* Siebold and Zucc] (Log) is an iridoid glycoside isolated from *Corni fructus* [99]. Log has received wide attention because of its extensive pharmacological effects, including anti-diabetes, neuroprotection, and anti-tumor activities. Studies have shown that these potential mechanisms include anti-oxidation, anti-inflammation, and anti-apoptosis, which regulate the transcription 3 (STAT3)/NF-κB, Janus kinase (JAK)/STAT3, and phosphatidylinositol-4,5-bisphosphate 3-kinase (PI3K)/protein kinase B (Akt) signaling pathways [100]. Some studies have found that perhaps Log can treat OA by inhibiting apoptosis. Yang et al. [58] evaluated the level of apoptosis, the expression of related factors and signal pathways using WB, PCR, and immunofluorescence in vivo and in vitro. They found that Log activates PI3K/Akt signal transduction, enhances the expression of Bcl2, and decreases the expression of Bax, thereby inhibiting apoptosis and exerting cartilage protection. However, their experiment had some limitations, and the specific mechanism between PI3K/Akt and apoptosis still needs to be discussed, which requires animal experiments. Therefore, further research is necessary to explore the specific mechanism of Log in treating OA, which will become an effective target for treating OA.

Generally, the absorption rate of Log in the body is very fast, but its pharmacokinetic properties are also affected by the different processed products of the herb [101]. The distribution of Log in different tissues is different. Li et al. [102] studied the tissue distribution of Log in rats with a single dose of 20 mg/kg using high-performance liquid chromatography technology. The results showed that Log was most distributed in the rats’ kidneys, followed by stomachs, lungs, and small intestines, and least distributed in their brains. Therefore, the correct route and dosage of administration should be selected according to the pharmacokinetic characteristics of Log. The metabolic process of Log is very complicated. Under the action of intestinal bacteria and enzymes in vivo, Log can generate various metabolites through deglycosylation, methylation, hydrogenation, hydroxylation, glucuronidation, and sulfation [103]. The pharmacokinetics of Log in its normal state are different from that in its pathological state. We should pay attention to the pharmacokinetic changes under pathological conditions, study the drug metabolism under pathological conditions, better understand the bioavailability and toxicity of drugs, strengthen the safety of drugs, and, finally, apply them to clinical treatment.

### 3.8. Andrographolide

Andrographolide [*Acanthaceae*; *Andrographis paniculata* (Burm.f.) Wall. ex Nees] (AG) is a natural antibiotic and the main active component of *Andrographis paniculata*. It can remove heat, expel toxins, relieve inflammation and pain, and effectively treat inflammatory diseases [104]. AG shows a wide range of biological activities, which has been a hot research field for many years [105]. AG plays a potential role in anti-inflammatory, anti-oxidative stress, and anti-apoptosis functions. Many studies have found that AG can alleviate OA by regulating apoptosis. Chen et al. [59] established an OA mouse model and made the mice take AG orally. The pathological changes of articular cartilage were evaluated via safranine O staining, the proliferation and apoptosis of chondrocytes were detected via CCK8 and flow cytometry, and the target of miRNA was evaluated via bioinformatics analysis and the luciferase reporter gene. Their results showed that cartilage degeneration improved and apoptosis was reduced. Subsequently, the expression levels of miR-27-3p in the proximal tibia of mice were measured, and they found that oral AG can reverse the downregulation of miR-27-3p induced via OA. MMP-13 is the direct target of miR-27-3p. Similarly, the primary chondrocytes isolated from mice were tested in vitro. Their results suggested that AG can prevent apoptosis by inhibiting miR-27-3p, thereby resisting OA. The research on the treatment of OA using AG is still in the primary stage, and the specific mechanism of action needs further exploration. This study found that AG may be used as a therapeutic drug for OA, which needs further verification in the future. Because AG can cause reproductive toxicity and renal toxicity [39,106], the toxicity of AG is mainly related to the concentration and time of administration, and long-term accumulation and rapid administration can cause the above adverse events. Therefore, before applying AG to the clinical treatment of OA, it needs to be further evaluated in clinical trials.

### 3.9. Geniposide

Geniposide [*Rubiaceae*; *Gardenia jasminoides* J.Ellis] (GE) is a natural compound derived from *Gardenia* with anti-inflammatory, anti-angiogenesis, anti-apoptosis, and other pharmacological effects [107]. GE has demonstrated multiple biological activities in treating brain, cardiovascular, liver, tumor, and other diseases [108]. In addition, GE can be used to treat OA, mainly through anti-inflammatory and anti-apoptotic effects [109]. Pan et al. [60] studied the therapeutic effect of GE on osteoarthritis in vivo and in vitro via WB, immunofluorescence, and histological detection. In vivo experiments showed that GE can alleviate the pathological process of OA in rats. In vitro, GE inhibits the expression of iNOS, COX-2, and MMP 13. At the same time, it promotes the expression of COL2A1 in IL-1β-stimulated rat chondrocytes. In addition, their study found that GE inhibits the expression of pro-apoptotic proteins such as Bax, Cyt c, and caspase 3 and increases the expression of anti-apoptotic protein Bcl2. These changes may be related to GE inhibiting the activation of the PI3K/Akt/NF-κB signaling pathway. Thus, GE has potential therapeutic value in the treatment of OA. However, the efficacy of GE compared with traditional drugs remains to be verified and whether the same effect on human OA chondrocytes would be observed requires further validation.

In addition, there are significant differences in the pharmacokinetics of GE in different modes of administration and different disease models. GE was absorbed rapidly after oral administration; the plasma concentration reached its peak after 1 h, and the GE was quickly eliminated from plasma within 12 h. Moreover, the absolute oral bioavailability of GE is low, about 9.67%. At the same time, after oral administration of GE, the study of tissue distribution in rats showed that the concentration of GE was highest in the kidneys and lowest in the brain [110]. Regarding the safety of GE, it was found that high-dose and continuous administration of GE can induce liver and kidney damage, but low-dose administration of GE has no toxic effect [111]. Therefore, many studies still need to be performed before GE can be used in the clinical treatment of OA patients.

### 3.10. Morroniside

Morroniside [*Cornaceae*; *Cornus officinalis* Siebold and Zucc] (MR), derived from the botanical drug *Cornus officinalis*, is an iridoid glycoside with many biological effects, such as anti-inflammatory, antioxidant, and anti-apoptotic activities [112]. MR can be used to treat OA by acting through the anti-apoptosis function. Yu et al. [61] pretreated OA mice with low and high doses of MR to evaluate the therapeutic effect of MR on cartilage degeneration. MR was found to attenuate the progression of OA mice and significantly inhibit osteophyte formation and hardening of subchondral bone. Moreover, MR downregulated the expression of caspase 1, inhibited the conduction of the NF-κB signaling pathway, and alleviated apoptosis. Therefore, MR prevents cartilage matrix degradation and reduces chondrocyte apoptosis by inhibiting NF-κB signaling. Yu et al. [61] used concentrations of 4 and 20 µg/kg and found that both low and high MR doses exert a protective effect on articular cartilage, but low doses of MR are more effective than high doses. It is possible that high-dose MR will activate other signal pathways at the same time, which will have harmful effects on cartilage. Cheng et al. [113] used concentrations of 10, 50, and 10,000 μg/kg to find that MR reduces chondrocyte apoptosis dose-dependently and has a protective effect on chondrocytes. These two different experimental conclusions may be caused by the difference between species (mice and rats) and the different methods of modeling OA. However, there are few studies on the treatment of OA using MR, and there is no clear conclusion on the effective dose of MR. The dose–effect relationship of MR should be further studied in the future.

### 3.11. Leonurine

Leonurine [*Lamiaceae*; *Leonurus sibiricus* L.] (LEO) is an active ingredient extracted from the leaves of *Herba leonuri*, which has many biological effects, such as anti-oxidation, anti-apoptosis, and anti-inflammation functions. LEO can treat many diseases by inhibiting apoptosis, such as heart disease [114], inflammatory bowel [115], and nervous system diseases [116]. LEO can also protect articular chondrocytes and alleviate OA through the anti-apoptosis effect. For example, Hu et al. [62] found, through PCR, ELISA, and WB, that LEO treatment decreases the mRNA and protein levels of Bax, increases the mRNA and protein levels of Bcl2, and inhibits the activation of MAPK and NF-κB in chondrocytes. LEO can play an anti-apoptosis role by regulating the NF-κB and MAPK signaling pathways in chondrocytes, which is of great value in preventing and treating OA. The results of the tissue distribution test in rats show that LEO is widely distributed in various tissues. The concentration of LEO was highest in the small intestine, followed by the liver and kidneys. However, the absolute oral bioavailability of LEO is very low, about 4.2% in rats, which may be related to the metabolic process of LEO in vivo [117]. Therefore, in the future, we should not only focus on the anti-apoptosis mechanism of LEO in the treatment of OA and provide a theoretical basis for the drug development of OA but also find suitable methods to improve the oral bioavailability of LEO and gradually carry out clinical research under the condition of ensuring dose safety.

### 3.12. Panax Notoginseng Saponins

*Panax notoginseng* saponins [*Araliaceae*; *Panax notoginseng* (Burkill) F.H.Chen] (PNS) are the main active components of *P. notoginseng*, and they have anti-apoptosis, anti-inflammation, and anti-oxidation functions. PNS can treat allergic rhinitis [118], nervous system diseases [119], and kidney diseases [120]. In addition, PNS can treat OA through an anti-apoptosis effect. Zhang et al. [63] studied the effect of PNS on the aging and apoptosis of osteoarthritis chondrocytes for the first time in vivo and in vitro using RT-qCPR, flow cytometry, immunofluorescence, and immunohistochemistry. In vivo experiments showed that PNS significantly inhibits the degradation of the PI3K-AKT-mTOR signaling pathway and COL2A1 in the OA rat model, thereby weakening cartilage destruction in OA. In vitro experiments showed that the expression level of anti-apoptosis protein Bcl2 in OA chondrocytes treated with PNS increased significantly. However, the expression levels of Bax and caspase 3 decreased; the phosphorylation of PI3K, AKT, and the mechanistic target of rapamycin (mTOR) decreased; and PNS affected the expression of these proteins in a concentration-dependent manner. In addition, PNS can prevent reductions in MMP and excessive mitochondrial permeability and significantly inhibit the aging and apoptosis of OA chondrocytes. Both in vivo and in vitro experiments proved that PNS can protect OA chondrocytes from aging and apoptosis by inhibiting the PI3K-AKT pathway.

In addition, studies have compared the pharmacokinetics of intramuscular injection and intravenous injection of PNS, and the results show that the main circulation forms of the two administration methods are basically the same in vivo. Although the detection rate of metabolites via intramuscular injection is low, their bioavailability is not much different [121]. Therefore, intramuscular injection and intravenous injection can be used as effective ways to administer PNS. Previous studies have discussed the effect of PNS in treating diabetes and stroke in clinical research [122,123], but the clinical research of PNS in treating OA is still relatively limited and the treatment evidence is not sufficient, which needs further experimental verification.

### 3.13. Shikonin

Shikonin [*Boraginaceae*; *Lithospermum erythrorhizon* Siebold and Zucc] (SK) is a bioactive red pigment and a traditional botanical drug isolated from flavonoid glucuronide. It has been used in traditional medical systems to treat various diseases with few side effects and is known for its multiple pharmacological potentials, such as anti-cancer, anti-thrombotic, and neuroprotective functions [124]. SK treats acute myocardial infarction [125], acute lung injury [126], and rheumatoid arthritis [127] by inhibiting apoptosis. Studies have also found that SK can treat OA through anti-apoptotic effects. PI3K/Akt is an essential pathway for SK to play an anti-apoptotic role in OA. Wang et al. [65] found, via ELISA, RT-PCR, WB, and cell viability testing, that SK pretreatment can improve the cell viability of IL-1β-stimulated rat chondrocytes, inhibit the PI3K/Akt signaling pathway, increase the expression of Bcl2, decrease the expression of Bax and caspase 3, inhibit the release of Cyt c from mitochondria to the cytoplasm, and reduce the permeability of chondrocytes’ mitochondria. Therefore, SK protects chondrocytes from IL-1β-induced apoptosis by inhibiting the mitochondrial apoptosis pathways and downregulating apoptosis factors such as caspase 3, playing a role in safeguarding articular cartilage. Fu et al. [64] explored the underlying mechanism of SK in OA rats. Their results showed that SK preconditioning significantly inhibits inducible nitric oxide synthase (iNOS), COX-2, and caspase 3 and increases Akt phosphorylation in the OA rat model. SK inhibits inflammation and apoptosis of chondrocytes by regulating the PI3K/Akt signaling pathway. These findings suggested that SK has therapeutic potential for OA and is a research direction for OA drug development. However, up to now, only the PI3K/Akt pathway has been found in the research on SK’s anti-OA capacityby inhibiting apoptosis. We believe that there must be other signal pathways or cytokines that play a role in the process of SK’s anti-OA capacity by inhibiting apoptosis, which needs further research and exploration in the future.

Oral or intragastric injection of SK does not produce toxicity or has little toxicity. Su et al. injected 200, 400, and 800 mg/kg shikonin into the stomach of adult rats, respectively, and then made continuous hematological and biochemical analysis, and no toxicity was found [128]. However, when SK is injected intraperitoneally and intravenously, great toxicity can be observed in the tested animals [129]. This may be due to the different absorption rate and exposed environmental conditions of drugs when they are administered through different routes. In addition, other factors such as metabolism, toxicity kinetics, and experimental uncertainty may also affect the toxicity level of SK in different routes of administration. Although oral administration of SK is relatively less toxic, it also has defects. Under oral administration of SK, the first-pass metabolism occurs together with a low intestinal absorption rate, which leads to a decrease in the bioavailability of SK. At present, there is no clinical study of SK in OA. It is hoped that the bioavailability of SK in vivo can be improved, its toxicity can be reduced, and its clinical research can be gradually expanded.

### 3.14. Stevioside

Stevioside [*Asteraceae*; *Stevia rebaudiana* (Bertoni) Bertoni] (SVS) is a natural diterpenoid glycoside, one of the natural sweeteners isolated from *Stevia* [130]. In terms of targeting OA, SVS has a variety of biological activities, such as anti-inflammatory [131], inhibiting cartilage degradation [131], and anti-apoptosis [66]. The anti-apoptosis mechanism of SVS in OA was discussed in this paper. Using CCK8, RT-PCR, WB, immunofluorescence, and various histological tests, Cai et al. [66] found that SVS pretreatment increases the level of anti-apoptotic gene Bcl2 and decreases the level of pro-apoptotic genes Bax and caspase 3. At the same time, SVS partially inhibits the phosphorylation of p65, p38, ERK, and JNK, thereby reducing the activation of NF-κB and MAPK. Therefore, SVS inhibits the apoptosis of murine chondrocytes and alleviates OA progression by regulating the MAPK and NF-κB pathways. Their research represents the first study on the efficacy of SVS in an OA model. SVS also has some toxicity. Yodyingyuad et al. conducted experiments on rats to explore the safe concentration of SVS. The results showed that when SVS was ingested at less than 25 mg/kg every day, there was no toxicity in rats, but it did cause toxicity in rats above this concentration [132]. This concentration can also be used as a reference for the safe concentration of clinical treatment. In conclusion, there are few studies on SVS for OA treatment, and there is a lack of research papers for reference. Further studies are needed to explore its specific mechanism and provide theoretical support for clinical application.

### 3.15. Achyranthes Bidentata Polysaccharides

*Achyranthes bidentata* polysaccharide [*Amaranthaceae*; *Achyranthes bidentata* Blume] (ABP) is an active component of the flowering plant *A. bidentata*, which has anti-inflammatory and antioxidant effects and can promote the growth of neurons [133] and enhance immune function [134]. The existing research shows that ABP has low toxicity and can be used as an herbal prescription to treat OA. Through histological evaluation, fluorescence in situ hybridization, RT-PCR, WB, and flow cytometry, Fu et al. [67] found that ABP can promote the repair of articular cartilage in OA mice, regulate the expression of lncRNA NEAT1 and miR-377-3p, and inhibit endoplasmic reticulum stress in the articular cartilage of mice in vivo and in vitro. ABP can alleviate the expression of endoplasmic reticulum stress-related factors in OA through the lncRNA NEAT1/miR-377-3p pathway, prevent cell apoptosis, and prevent and treat OA. This is the only preclinical study on the treatment of OA using ABP for the last decade, which shows that research on the application of ABP in OA is limited. However, ABP is low in toxicity and safe enough to be used as an herbal prescription [135]. Therefore, further experimental studies on ABP are warranted to tap into its potential for OA treatment.

### 3.16. Engeletin

Engeletin [*Smilacaceae*; *Smilax glabra* Roxb] (ENG) is a natural derivative of *Smilax rhizomilax*, which widely exists in fruits and vegetables [136], has anti-inflammatory activity, and inhibits lipid peroxidation [137]. It can be used to treat many diseases with minimal side effects. Compared with other flowering substances, the application of ENG is not extensive, and further research on it is warranted. To date, only one study has examined the effects of treating OA using ENG. Wang et al. [68] isolated articular chondrocytes from rat knee joints, induced an OA model via TNF-α, established an OA rat model using anterior cruciate ligament transection, and treated samples using ENG. Through WB, qPCR, Annexin V-FITC/PI, and flow cytometry, they found that ENG can maintain MMP, inhibit ROS production, alleviate chondrocyte apoptosis, and play a role in cartilage protection by upregulating Bcl2 expression and inhibiting Bax expression. This cartilage protection works by activating the erythroid 2-related factor 2 (Nrf2) pathway and inhibiting NF-κB and MAPK pathways. However, this study has some limitations. Firstly, this study did not clarify the interaction between MAPK and the NF-κB signaling pathway. Secondly, the potential mechanism of ENG has not been clarified via in vivo research, and it is suggested to evaluate it using immunohistochemistry. Nevertheless, this study proves that ENG may be a potential therapeutic drug for OA. There are few studies on the application of ENG in the treatment of diseases, and its pharmacokinetics, toxicity, and metabolic process are still unclear. In future experimental research, we should conduct in-depth research on ENG and further explore the mechanism of ENG in OA.

### 3.17. Baicalein

Baicalein [*Lamiaceae*; *Scutellaria baicalensis* Georgi] (Bai) is a kind of flavonoid with biological activity, which has many biological activities such as anti-inflammatory, anti-apoptosis, and anti-cancer. It can be used in the treatment of cancer, heart disease, and OA [138]. Yi et al. [54] predicted the action target of Bai through network pharmacology, located the target gene related to OA, and determined the potential anti-OA target of Bai. Then, the effect and potential mechanism of Bai on OA were verified using RT-PCR, WB, and various histological detection methods in the knee joints of OA mice. Through the combination of network pharmacology and experimental verification, it is found that Bai reduces the mRNA and protein expression of apoptosis-related factors BAX, BCL 2, and caspase 3 in chondrocytes, which means that Bai can alleviate cartilage injury and delay the progress of OA by inhibiting chondrocyte apoptosis. Unfortunately, this study did not analyze the mechanism of Bai for alleviating OA by affecting apoptosis-related pathways in vitro. Li et al. [55] detected the mRNA and protein expression of apoptosis factor via qRT-PC and WB, cell viability via CCK8, apoptosis via TUNEL staining and flow cytometry, and cartilage morphology via histological staining. In vivo and in vitro studies have found that Bai can alleviate OA by improving subchondral bone remodeling. Further research shows that Bai can regulate subchondral bone remodeling by inhibiting the differentiation and proliferation of osteoblasts and inducing apoptosis of osteoblasts. Different concentrations of Bai may have different effects on the regulation of osteoblast differentiation. They found that Bai effectively inhibited osteoblast differentiation at higher concentrations (10–50 μmol/L). In addition, Zhang et al. [56] used RT-PCR, WB, TUNEL staining, and histological methods to detect human articular chondrocytes and rat articular chondrocytes intervened using Bai. The results showed that Bai reduced the production of NO, the expression of caspase 3 and caspase 8, and inhibited chondrocyte apoptosis and cartilage degradation in a concentration-dependent manner. Therefore, the administration of Bai may represent a novel therapeutic approach in OA. However, low bioavailability and first-pass metabolism in vitro are important problems that Bai needs to solve in the process of treating diseases. Some technical means are needed to improve the pharmacokinetics of Bai and improve its use in the treatment of OA.

### 3.18. Genistein

Genistein [*Fabaceae*; *Genista* Linn.] (GE) is an isoflavone compound, which is abundant in soybeans, and has anti-inflammatory, anti-apoptosis, and anti-angiogenesis effects. Its extensive pharmacological properties can be used to prevent and treat many diseases such as heart disease, nonalcoholic fatty liver, and OA [139]. Zou et al. [57] found, through WB, ELISA, and flow cytometry, that GE can inhibit the release of TNF-α and IL-1β in OA models, thus reducing chondrocyte apoptosis and slowing down cartilage degeneration in a dose-dependent manner. Therefore, GE can be used as a suitable drug to treat OA by preventing cartilage degeneration and chondrocyte apoptosis. In addition, GE is also shown to be anti-inflammatory and inhibit cartilage degradation in OA. Some studies have found that a low dose (5–15 mg/kg/day) of GE has no obvious side effects on patients, but the safety of high-dose GE is still uncertain [140]. However, at present, there are few studies on the treatment of OA using GE, and the specific mechanism of action is not clear. The appropriate dose of GE for OA has not been studied, so we need to further study the potential mechanism and appropriate dose of GE for OA.

### 3.19. Epigallocatechin-3-gallate

Epigallocatechin-3-gallate [*Theaceae*; *Camellia sinensis* (L.) Kuntze] (EGCG) is a flavonoid, which is the main component of green tea. It has antioxidant, anti-inflammatory, and anti-aging effects and has many biological functions, such as preventing and treating diabetes, cancer, and heart disease [141]. Many studies have shown that EGCG has an anti-apoptosis effect on OA. Huang et al. [44] first ruled out the influence of EGCG on liver function and renal function in the experiment and then studied the role of EGCG in OA. They found that injecting EGCG into the joints of OA rats reduced cartilage degradation, and EGCG could regulate the progress of OA, reduce joint inflammation, and prevent chondrocyte apoptosis by promoting autophagy. In addition, Yang et al. [43] studied human OA chondrocytes and clinical samples and explored the mechanism of EGCG in OA via bioinformatics detection, qRT-PCR, WB, and flow cytometry. The results showed that EGCG upregulated PTEN by downregulating miR-29b-3p and inhibited inflammatory reactions, ECM degradation, and apoptosis. However, the regulation of the PTEN/miRNA-29b pathway via EGCG may also involve other regulatory pathways, which need further study. Although this study discussed the protective and therapeutic effects of EGCG on OA, there are some limitations in this study, such as the small sample size of clinical cases and the lack of animal experiments and clinical studies. Therefore, in the future, we should expand the sample size of clinical cases, increase animal experiments and clinical studies, and further explore the role and toxicity of EGCG in OA. Therefore, although some studies have proved the beneficial role of EGCG in OA, there are still some limitations. The relevant experimental research is not scientific and rigorous, and it is not enough to be applied to clinical treatment, so further exploration is needed. In addition, the bioavailability of EGCG is low after oral administration. The main reasons are decreased pharmacokinetics, limited biological distribution, first-pass metabolism, decreased accumulation in related tissues, and low targeting effectiveness [142]. Only by solving these problems can we better study the effect of EGCG on OA in clinical settings and provide safer and more scientific phytochemicals for the treatment of OA.

## 4. Clinical Application of Phytochemicals in the Treatment of OA

Phytochemicals are safe and effective drugs for alleviating OA. By studying the mechanism of phytochemicals in OA, we can identify the effective components and provide theoretical and practical bases for the alleviation of OA. Many preclinical studies have found that phytochemicals can alleviate OA by inhibiting apoptosis, and some studies have extracted effective components from plants and made them into drugs, which have been applied in clinical settings and proved to have good curative effects. For example, Farpour et al. [143] evaluated 60 patients with OA and found that *Harpagophytum procumbens* relieved pain and improved the function of patients with mild KOA in a short time. Huseini et al. [144] conducted experiments on patients with OA aged 50–70 years and determined the therapeutic effect of *Nigella sativa* in OA using the WOMAC score, VAS score, and patient satisfaction score. The results showed that *Nigella sativa* relieved pain and reduced OA symptoms. SheaFlex70 is an extract abundant in *Vitellaria paradoxa*. Cheras et al. [145] conducted a clinical experiment on 89 patients with OA given SheaFlex70. A 15-week randomized, controlled experiment was conducted to detect inflammatory markers, cartilage degradation markers, etc. SheaFlex70 reduced the expression of OA biomarkers. Continuous intake of *Psidium guajava* can also relieve knee pain in patients with OA and, thus, can be used to prevent the disease. Combining two or more phytochemicals can also have a good therapeutic effect on patients with OA. Rondanelli et al. [146] administered *Zingiber officinale* and *Echinacea angustifolia* extracts to patients with OA to determine their roles in inflammation and pain. The results showed an improved knee joint score, VAS, SF-36, and so on.

Some phytochemicals have not been widely used in clinical settings because of their low bioavailability, rapid metabolism, and uncertain safety. But, these problems are gradually being solved, such as the use of nanotechnology-based drug delivery systems. Based on the interaction between phytochemicals and substances in individuals at the molecular level, we can use the corresponding phytochemicals to alleviate OA given the definite therapeutic targets. At the cellular and molecular levels, phytochemicals will specifically select cytokines or pathways to combine to alleviate the pathological change mechanism of OA and prevent the disease. Every phytochemical has its unique function and efficacy. We can provide individualized treatment to patients according to their clinical symptoms and signs. In clinical intervention, the vast majority of phytochemicals can relieve pain and improve the knee joint function of patients with OA, as shown in Table 2. Hence, phytochemicals may be used as an effective drug to alleviate OA in clinical practice. It is worth further study and discussion.

## 5. Summary and Prospect

With the development of the aging population, the incidence of OA increases, which will not only seriously affect people’s lives but also bring a heavy burden to society [170]. At present, the main treatment methods for OA include surgery, exercise therapy, physical factor therapy, and drug therapy [171]. If the patient’s condition is serious, a large amount of articular cartilage is lost and the joint function declines, which seriously affects daily life activities. If conservative treatment is ineffective, joint replacement surgery is needed. However, the surgical trauma is large, the service life of the replaced artificial joint is limited, and the operation cost is high. Exercise therapy can enhance joint stability and muscle strength, etc., and is suitable for the chronic stage of OA, but the course of treatment is long, which requires the continuous participation and cooperation of patients. Physical factor therapy can improve local blood circulation, diminish inflammation, relieve pain, and improve joint function, etc., which is suitable for both acute and chronic stages of OA. But, generally, as an adjuvant therapy, it is difficult to completely cure OA patients only using physical factor therapy. For patients with mild to moderate OA, drug therapy is more widely used. The drug treatment of OA includes non-steroidal anti-inflammatory drugs and other traditional clinical drugs. However, traditional clinical drug treatment has some limitations in terms of safety and effectiveness. Long-term use has side effects to patients’ bodies and may cause serious harm to gastrointestinal function [172]. Many scholars at home and abroad have begun studying a new class of drugs to alleviate OA: phytochemicals. In recent years, the use of phytochemicals to prevent and treat OA has gradually become popular and attracted wide attention. As a natural compound, phytochemicals have remarkable pharmacological effects. They can be applied to alleviate OA and exert a protective effect on the liver and gastrointestinal tract. Phytochemicals are mostly water-soluble substances that can be quickly absorbed in the body, so they are worthy of wide clinical application [173]. Several studies have explored the mechanism of phytochemicals in OA through animal, cell, and clinical experiments.

Apoptosis is one of the important factors in the pathogenesis of OA. The inhibition of apoptosis plays a positive role in protecting articular cartilage in the pathological process of OA, and the inhibition of apoptosis has broad clinical application prospects as a therapeutic target for OA [174]. The targeted application of drugs to inhibit chondrocyte apoptosis is expected to provide additional options for the clinical treatment of OA. Phytochemicals have a specific anti-OA ability, mainly achieved by inhibiting apoptosis-related pathways and factors [71]. By summarizing the apoptosis mechanism of phytochemicals against OA, it is found that an appropriate dosage of phytochemicals can affect apoptosis factors such as Bax, Bcl2, and the caspase family by regulating apoptosis-related signal pathways such as NF-κB, ERK1/2, AKT/mTOR, PI3K/AKT, MAPK, and AMPK; non-coding RNA; or some cytokines and, finally, inhibit apoptosis against OA. As a result of the low side effects, high cost-effectiveness, and broad applicability of phytochemicals, an increasing number of studies are examining various phytochemicals for the prevention and treatment of OA. Thus, phytochemicals have become a vital source for developing novel OA therapies. For some phytochemicals with low bioavailability, poor water solubility, and high toxicity, nanotechnology-based drug delivery systems can be used to significantly improve them and maintain their stable release. Therefore, we should explore more effective delivery systems of various natural and synthetic phytochemicals for their targeted and specific delivery as soon as possible. In addition, it is necessary to explore new ways of synthesizing or producing phytochemicals, clarify their biomolecular targets, and evaluate their clinical potential.

However, research on the protective effect of phytochemicals on OA is still in the preclinical stage. Most of the research is basic, and clinical studies are relatively few. Moreover, preclinical research is not enough to fully explain that this phytochemical can be used in clinic settings, and its clinical efficacy and safety are not clear. In addition, the existing clinical research experiment design is relatively simple, and many of them can only prove whether a certain phytochemical is effective or not; comparative research on cytotoxicity, efficacy, and safety between phytochemicals and traditional clinical drugs is lacking. Unfortunately, our review has the limitation that there is a risk of obtaining false-positive results. However, under the background of developing natural products with targeted OA, our review is based on the available evidence to summarize and critically evaluate phytochemicals that resist OA by inhibiting apoptosis and explain, in detail, the specific mechanism through which these phytochemicals play an anti-apoptosis role, which provides a basis for further research and development. Therefore, we call on researchers to carry out systematic experimental design and experimental analysis in future research and establish an effective evaluation system. Let the research on the efficacy of phytochemicals in OA have more credibility, scientificity, and safety and benefit patients with OA more effectively.

## Figures and Tables

**Table 1:** Phytochemicals inhibit apoptosis against OA in vitro/in vivo.

Type	Phytochemical	Plant Species/Family	Plant Sources	Model	Route of Treatment	Dosage Range	The optimal Active Concentration	Duration	Signal Pathways/Mechanisms	References
Polyphenols	Curcumin	*Curcuma longa* L., *Zingiberaceae* Martinov	*Curcuma longa*	In vitro, human chondrocytes	Cell seeding	1, 2, 5, and 10 μM	5 μM	10 days	Sox9/NF-kB	[31]
				In vivo, ACLT and MMx surgery rats In vitro, SNP-induced rat chondrocytes	In vivo, intra-articular injection In vitro, cell seeding	In vivo, 20 and 40 μM In vitro, 1–30 μM	In vivo, 40 μM In vitro, 10 μM	In vivo, 10 weeks In vitro, 2 h	NF-κB/HIF-2α	[32]
				In vivo, surgery mice In vitro, IL-1β-induced murine chondrocytes	/ In vitro, cell seeding	/ In vitro, 1 and 5 μM	/ /	/ In vitro, 48 h	miR-124/NF-kB miR-143/ROCK1/TLR9	[33]
				In vitro, SNP-induced rabbit chondrocytes	Cell seeding	5, 10, and 20 μM	20 μM	24 h	Inhibit mitochondrial-dependent apoptosis pathway and restore the balance between synthesis and degradation of extracellular matrix.	[34]
				In vitro, human chondrocytes	Cell seeding	0, 5, 10, 20, and 40 μmol/L	40 μmol/L	24 h	Downregulate the expression of MMP3 and regulate apoptosis.	[35]
				In vitro, IL-1β-induced rat chondrocytes	Cell seeding	5, 10, 15, and 20 μM	10 μM	4 h	ERK1/2	[36]
				In vivo, HFD rats	Intra-articular injection	200 and 400 μg/kg	200 μg/kg	4 weeks	E2F1/PITX1, AKT/mTOR	[37]
	Resveratrol	*Vitis vinifera* L., *Vitaceae* Juss	Grape leaves	In vivo, MCL surgery rabbits	Intra-articular injection	50, 20, and 10 μmol/kg	50 μmol/kg	2 weeks	Reduce the production of NO in chondrocytes	[38]
				In vivo, HFD mice	Oral administration	5, 22.5, and 45 mg/kg	45 mg/kg	2 weeks	Reduce the degradation of type II collagen and inhibit chondrocyte apoptosis	[39]
				In vitro, IL-1β-induced murine chondrocytes	Cell seeding	5, 10, 20, 50, and 100 μM	10 μM	12 h	COX-2/NF-κB	[40]
				In vitro, human chondrocytes	Cell seeding	10 μM	10 μM	48 h	Sirt1, Wnt/β-catenin	[41]
	Chicoric acid	*Echinacea purpurea* (L.) Moench., *Asteraceae* Bercht. and J. Presl	*Echinacea purpurea*	In vivo, MIA-induced rats In vitro, TNF-α-induced human chondrocytes	In vivo, oral administration In vitro, cell seeding	In vivo, 16 and 32 mg/kg In vitro, 0, 2.5, 5, 10, 20, 40, and 80 μM	In vivo, 32 mg/kg In vitro, 20 μM	In vivo, 4 weeks In vitro, 24 h	PI3K/AKT, NF-κB	[42]
Flavonoids	Epigallocatechin-3-gallate	*Camellia sinensis* (L.) Kuntze, *Theaceae*	green tea	In vitro, IL-1β-induced human chondrocytes	Cell seeding	20 and 50 μM	50 μM	2 h	PTEN/miRNA-29b	[43]
				In vivo, ACL surgery rats	Cell seeding	40 μM	40 μM	5 weeks	/	[44]
	Icariin	*Epimedium brevicornu* Maxim., *Berberidaceae* Juss	Epimedium	In vitro, TNF-α-induced rat chondrocytes	Cell seeding	0, 3, 5, 7, 10, and 20 μM	10 μM	24 h	NF-κB	[45]
				In vitro, collagenase-induced rat chondrocytes	Cell seeding	10, 20, and 40 ng/mL	10 ng/mL	7 days	TDP-43	[46]
				In vitro, oxygen-, glucose-, and serum-deprivation-induced rabbit bone-marrow-derived mesenchymal stem cells	Cell seeding	0.1, 1, and 10 μM	10 μM	24 h	MAPK	[47]
				In vitro, IL-1β-induced human and rat chondrocytes	Cell seeding	0, 10, 20, and 30 μM	30 μM	48 h	lncRNA CYTOR	[48]
	Quercetin	/	Plant extracts such as rutin, glycoside, isoglycoside, anisidine, luteolin, wintersweet glycoside, etc.	In vivo, DMM surgery rats In vitro, IL-1β-induced rat chondrocytes	In vivo, intraperitoneal injection In vitro, cell seeding	In vivo, 50 mg/kg In vitro, 0–100 μM	In vivo, 50 mg/kg In vitro, 25 μM	In vivo, 12 weeks In vitro, 24 h	SIRT1/AMPK	[49]
				In vivo, DMM surgery rats In vitro, IL-1β-induced rat chondrocytes	In vivo, intra-articular injection In vitro, cell seeding	In vivo, 8 μM In vitro, 0–16 μM	In vivo, 8 μM In vitro, 8 μM	In vivo, 6 weeks In vitro, 24 h	Regulating the polarization of synovial macrophages to M2 macrophages	[50]
				In vitro, IL-1β-induced rat chondrocytes	Cell seeding	0–600 μM	100 μM	24 h	MAPK	[51]
				In vivo, ACL surgery rats In vitro, IL-1β-induced rat chondrocytes	In vivo, intraperitoneal injection In vitro, cell seeding	In vivo, 49.5 and 99 nM In vitro, 8 μM	In vivo, 99 nM In vitro, 8 μM	In vivo, 12 weeks In vitro, 2 h	IRAK1/NLRP3	[52]
	Isoliquiritigenin	*Glycyrrhiza glabra* L., *Fabaceae* Lindl	*Glycyrrhiza uralensis*, *Cicer arietinum*, *Dalbergia sericea*, *Dalbergia stevensonii*, and *Sophora tomentosa*	In vivo, ACLT surgery mice In vitro, IL-1β-induced murine chondrocytes	In vivo, intraperitoneal injection In vitro, cell seeding	In vivo, 10, 20, and 40 mg/kg In vitro, 2.5, 5, 10, 20, and 40 μmol/l	In vivo, 40 mg/kg In vitro, 10 μmol/l	In vivo, 8 weeks In vitro, 1 h	NF-κB	[53]
	Baicalein	*Scutellaria baicalensis* Georgi, *Lamiaceae*	*Scutellaria baicalensis*	In vivo, DMM surgery mice	Intragastric injection	50 mg/kg	50 mg/kg	8 weeks	/	[54]
				In vivo, DMM surgery rats In vitro, osteogenic differentiation	In vivo, intra-articular injection In vitro, cell seeding	In vivo, 1 mg In vitro, 0, 2.5, 5, 10, 20, and 50 μmol/L	In vivo, 1 mg In vitro, 10, 20, and 50 μmol/L	In vivo, 20 weeks In vitro, 24 h	/	[55]
				In vitro, IL-1β- and TNF-α-induced human chondrocytes	Cell seeding	0, 5, 10, 25, 50, and 100 μM	50 μM	0, 12, 24, 36, and 48 h	NF-κB	[36]
				In vitro, IL-1β-induced murine chondrocytes	Cell seeding	0, 5, 20, and 50 μM	20 and 50 μM	2 h	/	[56]
	Genistein	*Genista* Linn., *Fabaceae*	soybean	In vivo, ACL surgery rats In vitro, IL-1β-induced human chondrocytes	In vivo, intragastric injection In vitro, cell seeding	In vitro, 20 mg/kg In vitro, 25, 50, and 100 μg/mL	In vitro, 20 mg/kg In vitro, 100 μg/mL	In vitro, 6 weeks In vitro, 72 h	/	[57]
Terpenoids	Loganin	*Cornus officinalis* Siebold and Zucc., *Cornaceae* Bercht. and J. Presl	*Flos lonicerae*, *Cornus mas* L., and *Strychnos nux vomica*	In vivo, ACL surgery rats In vitro, IL-1β-induced rat chondrocytes	In vivo, subcutaneous injection In vitro, cell seeding	In vivo, 20 mg/kg/d In vitro, 0–20 μM	In vivo, 20 mg/kg/d In vitro, 10 μM	/ In vitro, 2 h	PI3K/Akt	[58]
	Andrographolide	*Andrographis paniculata* (Burm.f.) Wall. Ex Nees., *Acanthaceae* Juss	Stems and leaves of the botanical drug andrographis paniculata	In vivo, ACLT surgery mice	Oral administration	50 mg/kg	50 mg/kg	12 weeks	miR-27-3p/MMP13	[59]
	Geniposide	*Gardenia jasminoides* J.Ellis., *Rubiaceae* Juss	Gardenia	In vivo, MMT surgery rats In vitro, IL-1β-induced rat chondrocytes	In vivo, intraperitoneal injection In vitro, cell seeding	In vivo, 10 mg/kg In vitro, 0, 2.5, 5, 10, and 25 μM	In vivo, 10 mg/kg In vitro, 10 μM	In vivo, 8 weeks In vitro, 24 h	PI3K/Akt, NF-κB	[60]
	Morroniside	*Cornus officinalis* Siebold and Zucc., *Cornaceae* Bercht. And J. Presl	Dry flower buds of *Lonicera japonica Thunb.*	In vivo, DMM surgery mice In vitro, IL-1β-induced murine chondrocytes	In vivo, intra-articular injection In vitro, cell seeding	In vivo, 4 and 20 μg/kg In vitro, 20 and 100 μg/mL	/	In vivo, 8 and 12 weeks In vitro, 12 h	NF-κB	[61]
Small molecules compounds	Leonurine	*Leonurus sibiricus* L., *Lamiaceae* Martinov	Leaves of *Leonurus sibiricus* L. or whole grass of *Leonurus heterophyllus* Sweet.	In vitro, IL-1β-induced rat chondrocytes	Cell seeding	0–40 μM	5 μM	24 h	NF-κB, MAPK	[62]
	*Panax notoginseng* saponins	*Panax notoginseng* (Burkill) F.H.Chen., *Araliaceae* Juss	*Panax notoginseng*	In vivo, DMM surgery rats In vitro, TNF-α-induced rat chondrocytes	In vivo, intra-articular injection In vitro, cell seeding	In vivo, 100 and 200 mg/kg In vitro, 100 and 200 μg/mL	/ In vitro, 200 μg/mL	In vivo, 8 weeks In vitro, 24 h	PI3K-AKT-mTOR	[63]
	Shikonin	*Lithospermum erythrorhizon* Siebold and Zucc., *Boraginaceae* Juss	*Arnebia euchroma*	In vivo, ACLT surgery rats	Intra-articular injection	10 mg/kg	10 mg/kg	4 days	PI3K/Akt	[64]
				In vitro, IL-1β-induced rat chondrocytes	Cell seeding	0–8 μM	4 μM	2 h	PI3K/Akt	[65]
	Stevioside	*Stevia rebaudiana* (Bertoni) Bertoni., *Asteraceae* Bercht. and J. Presl	Stevia	In vivo, DMM surgery mice In vitro, IL-1β-induced murine chondrocytes	In vivo, oral administration In vitro, cell seeding	In vivo, 100 mg/kg In vitro, 0–200 μg/mL	In vivo, 100 mg/kg In vitro, 100 μg/mL	In vivo, 8 weeks In vitro, 24 h	NF-κB, MAPK	[66]
	*Achyranthes bidentata* polyscharides	*Achyranthes bidentata* Blume., *Amaranthaceae* Juss	*Achyranthes bidentata*	In vivo, ACLT surgery mice In vitro, thapsigargin-induced murine chondrocytes	In vivo, gavage In vitro, cell seeding	In vivo, 8 mg/kg/d In vitro, 0, 10, 25, 50, 100, and 150 μg/mL	In vivo, 8 mg/kg In vitro, 100 μg/mL	In vivo, 2 weeks /	lncRNA NEAT1/miR-377-3p	[67]
	Engeletin	*Smilax glabra* Roxb., *Smilacaceae* Vent	*Smilax glabra* Roxb	In vivo, ACLT surgery rats In vitro, TNF-α-induced rat chondrocytes	In vivo, intra-articular injection In vitro, cell seeding	In vivo, 50 μg/100 μL In vitro, 0–160 μM	In vivo, 50 μg/100 μL In vitro, 10 and 20 μM	In vivo, 8 weeks In vitro, 2 h	NF-κB, MAPK	[68]

**Table 2:** The role of plants in OA in clinical research.

Plant Name	Plant Species/Family	Dosage	Duration	Control	Design	Placebo	Function	References
*Harpagophytum procumbens*	*Harpagophytum procumbens* (Burch.) DC. ex Meisn., *Pedaliaceae* R. Br.	2 × 480 × 30 mg/d, pure compound	4 weeks	Positive control	Double-blinded RCT	No	Relieve pain and improve function	[143]
*Arctium lappa*	*Arctium lappa* L., *Asteraceae* Bercht. and J. Presl	3 × 2 g/d, powder	6 weeks	Negative control	RCT	Yes	Alleviate the progression of OA	[147]
*Psidium guajava*	*Psidium guajava* L., *Myrtaceae* Juss	1 g/d, powder	12 weeks	Negative control	Double-blinded RCT	Yes	Relieve pain	[145]
*Cissus quadrangularis*	*Cissus quadrangularis* L., *Vitaceae* Juss	3200 mg/d, powder	8 weeks	Negative control	Double-blinded RCT	Yes	Relieve pain	[148]
*Nigella sativa*	*Nigella sativa* L., *Ranunculaceae* Juss	2.5 mL/8 h, extract	4 weeks	Negative control	Double-blinded RCT	Yes	Relieve pain	[144]
*Vitellaria paradoxa*	*Vitellaria paradoxa* C. F. Gaertn., *Sapotaceae* Juss	3 × 750 mg/d, extract	15 weeks	Negative control	Double-blinded RCT	Yes	Alleviate the progression of OA	[149]
*Elaeagnus angustifolia*	*Elaeagnus angustifolia* L., *Elaeagnaceae* Juss	3 × 200 mg/d, extract	4 weeks	Positive control	Double-blinded RCT	No	Relieve pain and improve function	[150]
		300 mg/d and 600 mg/d, extract	7 weeks	Positive control	Double-blinded RCT	No	Relieve pain	[151]
		15 g/d, powder	8 weeks	Negative control	Double-blinded RCT	Yes	Alleviate the progression of OA	[152]
*Zingiber officinale*	*Zingiber officinale* Roscoe., *Zingiberaceae* Martinov	2 × 255 mg/d, extract	6 weeks	Negative control	Double-blinded RCT	Yes	Relieve pain and improve function	[153]
		37.5 mg/d, extract	4 weeks	No	One-group, pre-test–post-test, quasi-experimental pilot study	No	Relieve pain	[154]
		25 mg/d, extract	4 weeks	No	Uncontrolled multicenter study	No	Relieve pain	[146]
		1 g/d, pure compound	11 days	Negative control	Double-blinded RCT	Yes	Relieve pain	[155]
*Andrographis paniculata*	*Andrographis paniculata* (Burm.f.) Wall. ex Nees., *Acanthaceae* Juss	300 mg/d and 600 mg/d, extract	12 weeks	Negative control	Double-blinded RCT	Yes	Alleviate the progression of OA	[156]
*Pinus massoniana*	*Pinus massoniana* Lamb., *Pinaceae* Spreng. ex F. Rudolphi	1322 mg/d, pure compound	12 weeks	Negative control	Double-blinded RCT	Yes	Alleviate the progression of OA	[157]
*Curcuma longa*	*Curcuma longa* L., *Zingiberaceae* Martinov	2 × 500 mg/d, extract	12 weeks	Negative control	Double-blinded RCT	Yes	Relieve pain	[158]
		2 × 46.67 mg/d and 3 × 46.67 mg/d, extract	12 weeks	Negative control	Double-blinded RCT	Yes	Relieve pain	[159]
		500 mg/d, extract	4 months	Negative control	Double-blinded RCT	Yes	Alleviate the progression of OA	[160]
		1000 mg/d, extract	4 weeks	Positive control	A clinical study	No	Relieve pain	[161]
		2 × 500 mg/d, extract	21 days, 42 days	Negative control	Single-blinded RCT	Yes	Relieve pain	[162]
Curcumin	*Curcuma longa* L., *Zingiberaceae* Martinov	3 × 500 mg/d, extract	4 weeks	Positive control	RCT	No	Relieve pain	[163]
		2 × 500 mg/d, extract	4 weeks	Negative control	RCT	Yes	Relieve pain	[164]
		1500 g/d, extract	4 weeks	Positive control	A multicenter study	No	Relieve pain and improve function	[165]
Resveratrol	*Vitis vinifera* L., *Vitaceae* Juss	500 mg/d, powder	90 days	No	A noncontrolled clinical trial	No	Relieve pain and improve function	[166]
		500 mg/d, powder	90 days	Negative control, Positive control	Double-blinded RCT	Yes	Relieve pain and inflammation	[167]
		500 mg/d, powder	90 days	Negative control, Positive control	Double-blinded RCT	Yes	Relieve pain and improve function	[168]
*Artemisia annua*	*Artemisia annua* L., *Asteraceae* Bercht. and J. Presl	150 mg/d and 300 mg/d, extract	12 weeks	Negative control	Double-blinded RCT	Yes	Improve the pain, stiffness, and limited function of knee joint	[169]
